# Ablation rate after radioactive iodine therapy in patients with differentiated thyroid cancer at intermediate or high risk of recurrence: a systematic review and a meta-analysis

**DOI:** 10.1007/s00259-021-05440-x

**Published:** 2021-06-18

**Authors:** Michele Klain, Carmela Nappi, Emilia Zampella, Valeria Cantoni, Roberta Green, Leandra Piscopo, Fabio Volpe, Mariarosaria Manganelli, Elisa Caiazzo, Mario Petretta, Martin Schlumberger, Alberto Cuocolo

**Affiliations:** 1 Department of Advanced Biomedical Sciences, University Federico II, Naples, Italy; 2 IRCCS SDN, Naples, Italy

**Keywords:** Differentiated thyroid cancer, ^131^I, Meta-analysis, Intermediate risk, High risk

## Abstract

**Purpose:**

We performed a systematic review and a meta-analysis to investigate the successful ablation rate after radioiodine (RAI) administration in patients with differentiated thyroid cancer (DTC) at intermediate-high risk of recurrence.

**Methods:**

A comprehensive literature search of the PubMed, Scopus, and Web of Science databases was conducted according to the PRISMA statement.

**Results:**

The final analysis included 9 studies accounting for 3103 patients at intermediate-high risk of recurrence. In these patients, the successful ablation rates ranged from 51 to 94% with a 71% pooled successful ablation and were higher in intermediate (72%) than in high (52%)-risk patients. Despite the rigorous inclusion standards, a significant heterogeneity among the evaluated studies was observed. Higher administered RAI activities are associated with a lower successful ablation rate in the whole population and in the subgroup of high-risk patients. Furthermore, pooled recurrence rate in intermediate-risk patients achieving successful ablation was only 2% during the subsequent 6.4-year follow-up while the pooled recurrence rate was 14% in patients who did not achieve a successful ablation.

**Conclusion:**

In a large sample of 3103 patients at intermediate-high risk of persistent/recurrent disease, 71% of patients achieved a successful ablation. In these intermediate-risk patients, the probability of subsequent recurrence is low and most recurrence occurred in those with already abnormal findings at the first control.

**Supplementary Information:**

The online version contains supplementary material available at 10.1007/s00259-021-05440-x.

## Introduction

After total thyroidectomy with or without lymph node dissection for differentiated thyroid cancer (DTC), radioiodine (RAI) may be administered for 3 main goals [1]: remnant ablation (to facilitate the detection of recurrent disease by destroying post-operative remnants of non-tumoral thyroid tissue and to permit initial staging with a whole-body scan), adjuvant therapy (to decrease the risk of recurrence by destroying suspected, but unproven persistent disease), or therapy (to treat known persistent disease). Successful ablation (SA) can be assessed some months later by an undetectable serum thyroglobulin (Tg) level on L-T4 using a sensitive method and unremarkable findings at neck ultrasound [2–4].

The indication for post-operative RAI administration takes into account the American Thyroid Association (ATA) three-tiered risk system [1] that classifies patients as low, intermediate, or high risk of recurrence, although different patients’ classifications can also be used [5]. Whereas post-surgical RAI therapy is usually not indicated in low-risk patients, it is generally recommended in intermediate and high-risk patients. Intermediate-risk group includes patients with microscopic extra-thyroid extension, aggressive histology, vascular invasion, > 5 lymph node metastases (N1) with all N1 < 3 cm, multifocal papillary microcarcinoma with ETE or BRAFV600E mutation, and RAI-avid metastatic foci in the neck on the first whole-body RAI scan. High-risk patients show macroscopic ETE, incomplete tumor resection, biochemical or structural evidence of distant metastatic disease, any N1 > 3 cm, or follicular thyroid cancer with extensive vascular invasion. Although literature offers a huge armamentarium of data on survival benefit of RAI in intermediate-high risk patients, to our knowledge, a meta-analysis on SA rate in these patients [6, 7] has not yet been performed.

Therefore, we performed a systematic review and a meta-analysis to investigate the SA rate after RAI administration in patients with DTC at intermediate-high risk of recurrence.

## Methods

### Search strategy

This meta-analysis followed the Preferred Reporting Items for Systematic Reviews and Meta- Analyses (PRISMA) statement (see Supplementary Material for PRISMA Checklist) [8], and registered as 242,409 in the PROSPERO database (University of York, UK; http://www.crd.york.ac.uk/PROSPERO/). An English literature search was performed using the PubMed and Embase databases to identify articles published from January 2010 to June 2020. This search was restricted to data obtained in adults and was conducted using the following key words: “differentiated thyroid cancer” OR “DTC”, “thyroid neoplasm”, “prognosis”, “outcome”, “follow-up”, “radioactive iodine therapy” OR “RAI therapy”, “I-131 ablation”, “thyroglobulin” OR “Tg”.

### Study selection

The title and abstract of potentially relevant studies were screened for appropriateness before retrieval of the full article by two reviewers (L.P. and F.V.), and disagreements were resolved by consensus. The selected full-published reports were retrieved and the same reviewers independently performed a second-step selection based on the eligibility criteria; disagreements were resolved by consensus. In addition, the bibliographies of retrieved articles were manually reviewed for potential additional citations.

### Study eligibility and data extraction

Each study was initially identified considering journal, authors, and year of publication. To harmonize the predictors of interest, a study was considered eligible if all of the following criteria were met: (1) Data were available on age, gender, and administered RAI activity, histopathology, and extent of surgery. (2) The study presented data of adult subjects with differentiated thyroid cancer at intermediate or high risk of recurrence after RAI therapy; we excluded studies on low-risk patients only and we excluded the low-risk patients in studies that included both low and intermediate/high-risk patients [1]. Investigations that considered different risk classifications, such as the AJCC/TNM staging system [5], were included in the final analysis only if a detailed description of clinical and histopathological characteristics of patients was provided, allowing patient categorization with the ATA risk classification basis (e.g., studies only including patients having microscopic extra-thyroidal extension which are considered intermediate ATA risk or studies clearly providing data on patients with pT3-pT4 tumor or metastasis who were considered ATA high risk); (3) the study included at least 100 subjects; (4) follow-up after RAI therapy for at least 1 year; (5) the study provided data on SA after RAI therapy defined as absence of abnormal findings at neck ultrasonography and undetectable serum Tg in the absence of anti-Tg antibodies [2, 3]. In case of multiple studies reported from the same research group, potential cohort duplication was avoided by including only the study on the largest number of patients.

### Assessment of the methodological quality of studies

All studies were assessed for methodological quality using Joanna Briggs Institute (JBI) Prevalence Critical Appraisal Tool [9]. The criteria address the following issues: ensuring a representative sample, ensuring appropriate recruitment, ensuring an adequate sample size, ensuring appropriate description and reporting of study subjects and setting, ensuring data coverage of the identified sample is adequate, ensuring the condition was measured reliably and objectively, ensuring appropriate statistical analysis, and ensuring confounding factors/subgroups/differences are identified and accounted for. These questions can be answered with four possible responses: yes, no, unclear, or not applicable [9]. Two reviewers (V.C. and R.G.) evaluated the risk of bias in each eligible study, and disagreements between reviewers were resolved by consensus. The two reviewers completed the screening process independently. Disagreement in the process of answering questions was discussed until consensus was reached. A final decision of yes (favorable scenario, “ + ”), no (unfavorable scenario, “ − ”), or unclear (mixed scenario, “ + / − ”) was made by the reviewers after systematic discussion. If the answers to all the signal problems were “yes,” a low risk of bias was attributed to the study; if the answers to all the signal problems had one or more “no” or “unclear” values, an unclear risk of bias was used; if the answers to all the signal problems contained at least one “no” but no “yes” answers, a high risk of bias was attributed.

### Statistical analysis

We used a systematic analytic approach to compute the pooled SA rate after RAI therapy in patients with DTC from all eligible studies. All analyses were conducted using the logit transformed proportion as the effect size statistic and the inverse of the variance of the transformed proportion as study weight. The summary estimate and its confidence interval (CI) were back-transformed to proportions for easy interpretation. Heterogeneity of the included studies was examined by using the I-squared (I^2^) statistic, to reflect the percentage of total variation across studies [10], assigning adjectives of low, moderate, and high to I^2^ values of 25%, 50%, and 75%. According to the Cochrane handbook, I^2^ > 50% reflects a substantial heterogeneity [11]. Therefore, a random effect model was used to combine data in the meta-analysis [12]. The possibility of publication bias in the present study was examined by using Egger’s test [13]. Publication bias was graphically examined by the funnel plot and also formally assessed with the regression test of asymmetry described by Egger et al. [13]. A leave-one-out sensitivity analysis was also performed to evaluate if single study had a substantial influence on the overall effect size. A further sensitivity analysis was performed considering the studies with comparable administered RAI activities.

When it was feasible, we evaluated the pooled SA rate separately in DTC patients at intermediate and high risk of recurrence. To better understand the differences between these two categories, the SA relative ratio of intermediate-risk to high-risk patients was calculated.

We also evaluated the pooled recurrent disease rate in DTC patients who achieved SA when data were available. All analyses were performed using Stata, version 15.1 (StataCorp, College Station, TX). Two-sided *P* values < 0.05 were considered statistically significant.

#### Results

### Study selection

**Fig. 1:**
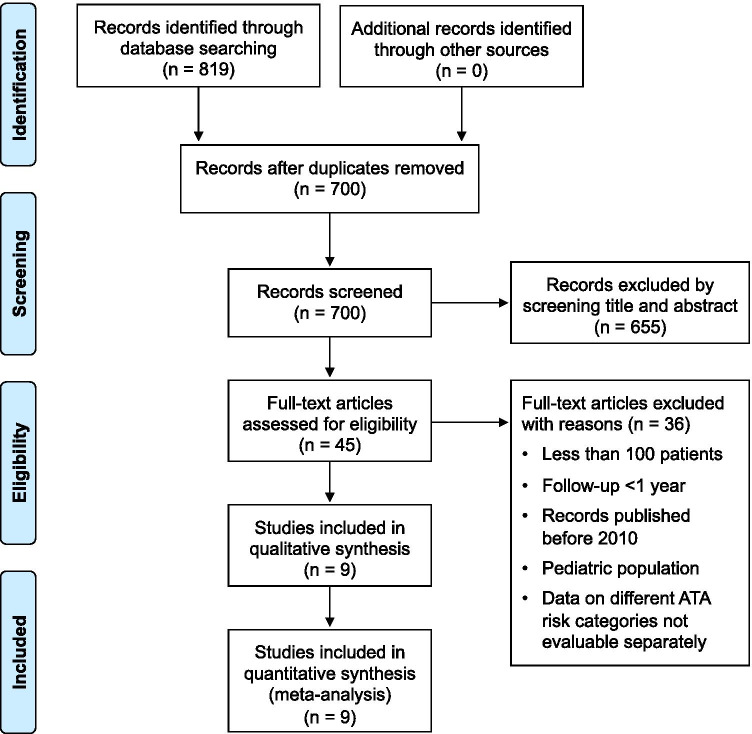
PRISMA flowchart illustrating the study selection process

The complete literature search is presented in Fig. 1. The initial search identified 819 potentially eligible citations. Among these, 119 were identified as duplicates and thus removed, leaving 700 records. The reviewers, after the evaluation of titles and abstracts of these studies, removed 655 citations. Then, each investigator blindly reviewed the full text of the remaining 45 articles, and 36 articles were excluded. The remaining 9 articles included a total of 6675 patients, 3103 of whom at intermediate-high risk of recurrence and were the basis of the present meta-analysis.

### Characteristics of the included studies

Demographic data and clinical characteristics of the 3103 patients included in the meta-analysis are detailed in Table 1 [14–22]. Study sample size ranged from 152 to 627 subjects. Mean follow-up was 4.7 ± 1.5 years. Seven of the 9 studies [14, 17–22] referred to the ATA risk classification and this permitted to define separate risk categories. One study [15] referred to AJCC/TNM staging system without mentioning the ATA risk classification. However, this investigation only included patients with small tumor size, extra-thyroidal extension, and no neck lymph node metastasis who were considered at intermediate risk in the separate analysis. In another study [16] only referring to the AJCC/TNM staging system, patients with pT3-pT4 tumors or metastasis were defined as high risk. Although one investigation [21] referred to ATA risk categories, SA rates were only available when considering both intermediate and high-risk patients together and this study was only considered for the overall analysis. Detailed information about neck dissection was only available in three studies [18, 20, 21]. Rosario et al. [18] reported 74% patients with lymph node metastases and Avram et al. [21] 67% patients with neck nodal metastases, and Llamas-Olier et al. [20] reported 95% patients with pN1 classification. Regarding preparation protocol, only two studies [20, 22] considered both rhTSH and LT4 withdrawal protocols, while the remaining seven studies included patients under LT4 withdrawal protocol.

**Table 1:** Demographic data and clinical characteristics of study population

	Patients included in the meta-analysis (*n*)	Age (years)	Women (%)	Extent of surgery	First RAI treatment activity (GBq)	Follow-up (years)	Risk classification	Intermediate risk (*n*)	High risk (*n*)
Caminha [14]	152	NA	NA	TT	3.63	13.7 ± 4.1	ATA	120	32
Han [15]	176	50 ± 9	96	TT	3.07	1	AJCC/TNM	176	−
Verburg [16]	600	NA	NA	TT	5.10	10	AJCC/TNM	−	600
Jeon [17]	627	NA	NA	TT	5.58	8	ATA	578	49
Rosario [18]	180	48	80	TT	1.11–5.5	1	ATA	180	−
Jeong [19]	204	44 ± 12	92	TT	2.92	2	ATA	204	−
Llamas-Olier [20]	389	51 ± 11	93	TT	3.99	1	ATA	389	−
Avram [21]	350	46 ± 16	66	TT	3.88	3.3 ± 1.9	ATA	350	
Kim [22]	425	NA	NA	TT	3.99	2.3	ATA	292	133

Values are expressed as mean ± standard deviation or median or as number (percentage) of subject

*TT* total thyroidectomy, *RAI* radioactive iodine

### Assessment of the methodological quality of the included studies

**Fig. 2:**
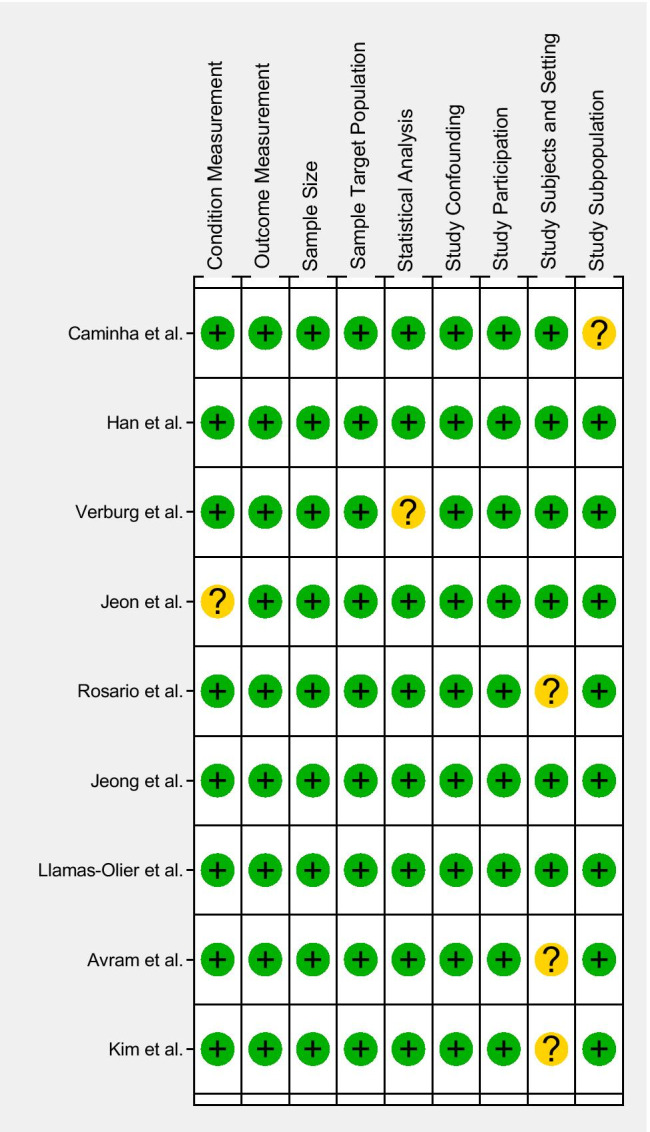
Methodological quality of the included studies assessed with JBI tool for risk of bias and applicability concerns. The green circle represents low risk of bias, the yellow circle unclear risk of bias, and the red circle high risk of bias

Figure 2 summarizes the quality assessment of the 9 included studies using Critical Appraisal Tools for use in JBI Systematic Reviews. The risk of bias was considered low overall. The domain that showed an unclear risk of bias was “study subjects and setting”. This result could be due to slightly different descriptions of patient characteristics among studies.

### Successful ablation rate in the overall population

**Fig. 3:**
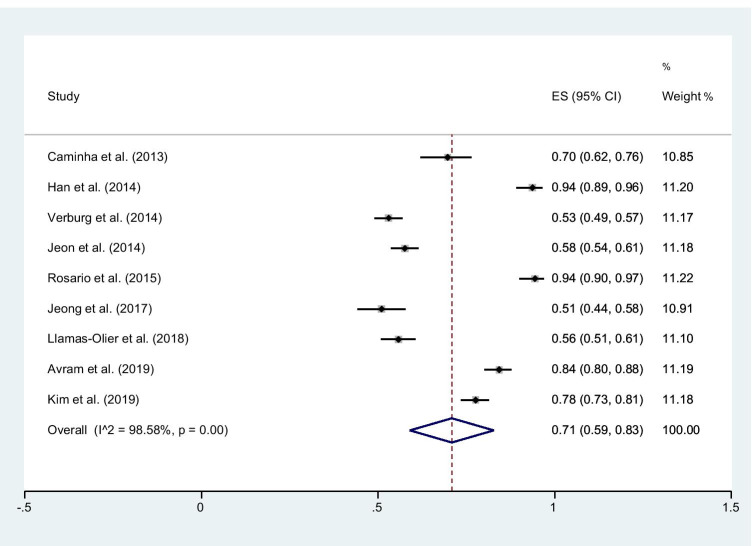
Forest plot for the successful ablation rate after RAI therapy. Horizontal lines represent 95% confidence interval of the point estimates. The diamond represents the pooled estimate (size of the diamond = 95% confidence interval). The solid vertical line represents the reference of no increased risk and the dashed vertical line represents the overall point estimate

The SA rate reported in the 9 studies ranged from 51 to 94% (Fig. 3), the pooled SA rate was 71% (95% CI, 59–83), and the heterogeneity was 98.58% (*P* < 0.001). The funnel plot indicates no publication bias (*P* = 0.16) among these studies (see Supplementary Material, Figure S1). The leave-one-out sensitivity analysis indicates that no single study had a substantial influence on the overall effect size (see Supplementary Material, Table S1).

A further sensitivity analysis was performed only considering studies with comparable administered RAI activities [14, 15, 19–22] (see Supplementary Material, Figure S2) showing an overall SA rate of 72% (95% CI, 59–85).

### Relative ratio of intermediate-risk patient over high-risk patient SA rates

The SA rate in intermediate-risk patients was reported in 7 studies [15, 16, 18–21, 23], including 1939 patients. It ranged from 51 to 94%, and the pooled SA rate was 76% (95% CI 61–86). The SA rate according to separate high-risk patients’ category has been reported in only 4 studies [14, 16, 17, 22] including 814 patients and ranged from 18 to 78%. The pooled SA rate was 62% (95% CI 44–77).

The relative ratio of intermediate-risk patient over high-risk patient SA rates was 1.22 (95% CI 1.05–1.42, *P* = 0.008) (see Supplementary Material, Figure S3).

Of note, the 350 patients at intermediate and high risk evaluated by Avram et al. [21] were not included in any subgroup analysis because data could not be evaluated in separate categories but only as a single group.

### Recurrent disease rate at in patients who achieved successful ablation

**Fig. 4:**
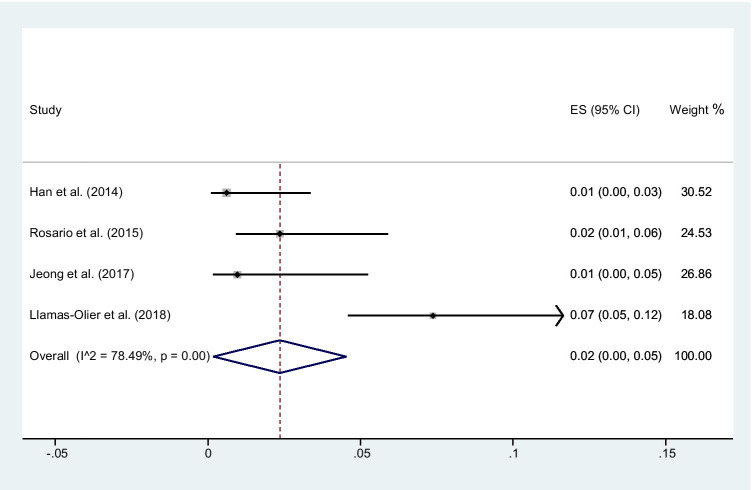
Forest plot for the persistent/recurrent disease rate at late follow-up in patients who achieved SA. Horizontal lines represent 95% confidence interval of the point estimates. The diamond represents the pooled estimate (size of the diamond = 95% confidence interval). The solid vertical line represents the reference of no increased risk and the dashed vertical line represents the overall point estimate

A late follow-up (mean 6.4 ± 1.4 years) was available in 4 studies [15, 18–20] including 656 intermediate-risk patients (Table 2). In one study [20], recurrence was defined as new evidence of biochemical or structural disease after any disease-free period; in the other 3 studies [15, 18, 19], recurrence was cytologically or histologically proven. Recurrence rate after SA ranged from 0 to 7% and the pooled recurrent disease rate was 2% (95% CI 0–5) (Fig. 4). The heterogeneity was 78.5% (*P* < 0.001). In contrast, recurrences were observed in 18/121 intermediate-risk patients who did not achieve an SA at the first control, with a pooled rate of 14%.

**Table 2:** Recurrence at late follow-up in patients with and without successful ablation at early follow-up

	Patients with recurrence/patients with successful ablation	Patients with recurrence/patients without successful ablation	Mean follow-up (years)
Han [15]	0/165	0/11	7.2
Rosario [18]	4/170	1/10	5
Jeong [19]	0/104	14/100	10
Llamas-Olier [20]	16/217	NA/172	3.5

Values are expressed as number of subjects

## Discussion

The present investigation refers to 9 studies accounting for 3103 patients at intermediate or high risk of recurrence. The main finding of our meta-analysis is a 71% pooled SA rate after RAI treatment and this was indeed higher in intermediate (72%) than in high (52%)-risk patients, as also confirmed by relative ratio of 1.22. This is well below the reported SA rates achieved in low-risk patients [23, 24], even with the use of relatively low activities (30 mCi) following rhTSH [24, 25]. Indeed, a sensitivity analysis performed only in studies with comparable administered RAI activities showed a pooled SA rate of 72%. This is consistent with the overall pooled SA rate of 71%.

Despite the rigorous inclusion standards, a significant heterogeneity among the evaluated studies was observed. The presence of heterogeneity in a meta-analysis is an expected issue [26]. This result could be explained by different specific end-points considered within each single investigation. It should be also taken into account that, as shown in Table 1, the overall population assessed in every single study referred to a more extensive patient group with heterogeneous clinical characteristics, while the evaluated population included in our analysis matched strict criteria suggesting that the investigations included in the current meta-analysis suffer from different degrees of bias across studies. To minimize the heterogeneity of data due to continuous and dynamic changes in the patient risk classification, imaging modality implementation, and overall diagnostic refinement, an interval time was also applied. Yet, we decided a priori to include only studies with at least 100 patients to increase the robustness of analysis and to have our findings more strength [27]. Still, some bias related to inclusion factors is present in some studies of the meta-analysis. It should be considered that clinical and methodological diversity always occur in a meta-analysis of observational studies; thus, statistical heterogeneity is almost inevitable [28, 29]. However, although we considered a limited time window for bibliography research from 2010 to 2020 in some studies evaluated [14–16], the period between DTC diagnosis and data analysis is very huge. Nevertheless, the meta-analysis method remains a powerful option to interpret multiple data coming from literature.

A late follow-up was available in 4 studies [15, 18–20] only considering intermediate-risk patients. In patients achieving SA at early follow-up, there was a pooled recurrence rate of 2%. In contrast, the pooled recurrence rate was 14% in patients who did not achieve an SA at early follow-up. These results are in close agreement with the prognostic value of the ATA classification, with the majority of recurrences occurring in patients with some abnormal findings at the first control [30]. Yet, data on high-risk patients were not available to perform a similar analysis in this category. Thus, the low recurrence rates after RAI treatment in both intermediate and high-risk patients are consistent with rationale of clinical use of ablation therapy.

## Conclusion

The present meta-analysis demonstrates that overall SA rate after RAI treatment in patients at intermediate-high risk of recurrence was 71% and it was higher in intermediate (72%) than in high (52%)-risk patients. Furthermore, pooled recurrence rate in patients achieving SA was only 2% further highlighting the very low probability of recurrence of disease once an SA has been obtained after RAI therapy. These data indeed underline the importance of using the concept of ongoing risk assessment that allows to modify the individual prognosis and follow-up strategy according to the results of each control.

## Supplementary Information

Below is the link to the electronic supplementary material.

Supplementary file1 Figure S1 Funnel plot for the successful ablation rate after RAI therapy. Each dot represents a study; the y-axis represents study precision (standard error of effect size) and the x-axis shows the effect size. Large studies appear toward the top of the graph and tend to cluster near the mean effect size. Small studies appear toward the bottom of the graph and are dispersed across a range of values since there is more sampling variation in effect size estimates. The outer dashed lines indicate the triangular region within which 95% of studies are expected to lie in the absence of biases and heterogeneity. S.E., standard error. (PDF 53 KB)

Supplementary file2 Figure S2 Forest plot for the successful ablation rate after RAI therapy in the six studies considering patients treated with comparable RAI activities. Horizontal lines represent 95% confidence interval of the point estimates. The diamond represents the pooled estimate (size of the diamond = 95% confidence interval). The solid vertical line represents the reference of no increased risk and the dashed vertical line represents the overall point estimate. (PDF 55.6 KB)

Supplementary file3 Figure S3 Forest plot of relative ratios of intermediate risk patients over high-risk patients SA rates. Horizontal lines represent 95% confidence interval of the point estimates. The diamond represents the pooled estimate (size of the diamond = 95% confidence interval). The solid vertical line represents the reference of no increased risk and the dashed vertical line represents the overall point estimate. (PDF 57.7 KB)

Supplementary file4 (DOCX 14.6 KB)

Supplementary file5 (DOC 66 KB)
