# Supplementary figures and images for: Ablation rate after radioactive iodine therapy in patients with differentiated thyroid cancer at intermediate or high risk of recurrence: a systematic review and a meta-analysis
Source: Eur J Nucl Med Mol Imaging. 2021 Jun 18;48(13):4437–44. doi: 10.1007/s00259-021-05440-x (PMC8566414; doi:10.1007/s00259-021-05440-x)

Funnel plot with pseudo 95% confidence limits

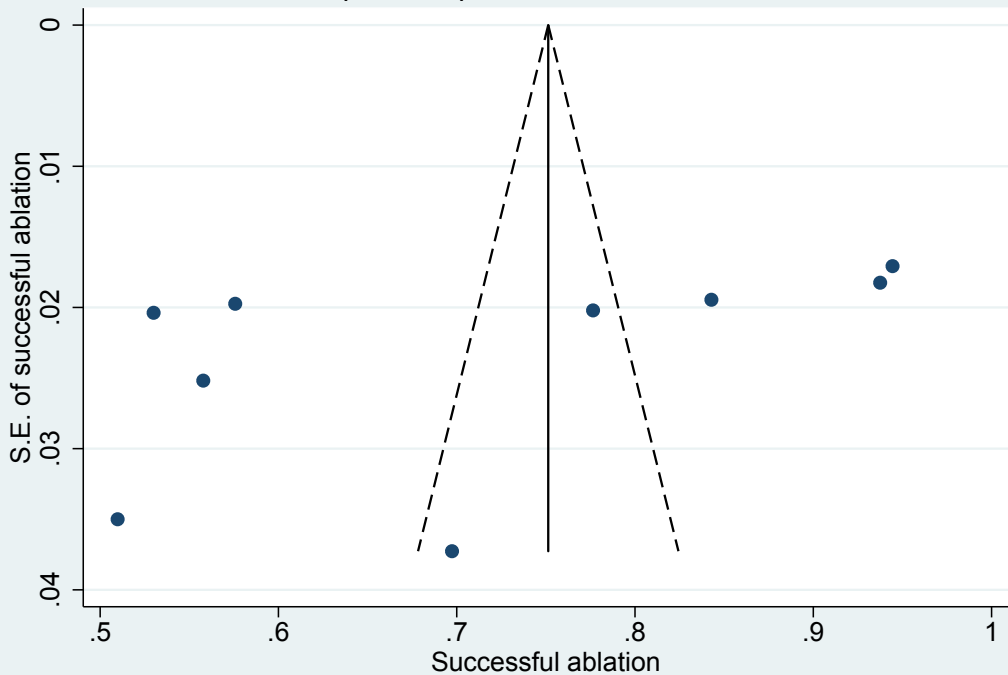

Supplement: Supplementary file 1 — Supplementary file1 Figure S1 Funnel plot for the successful ablation rate after RAI therapy. Each dot represents a study; the y-axis represents study precision (standard error of effect size) and the x-axis shows the effect size. Large studies appear toward the top of the graph and tend to cluster near the mean effect size. Small studies appear toward the bottom of the graph and are dispersed across a range of values since there is more sampling variation in effect size estimates. The outer dashed lines indicate the triangular region within which 95% of studies are expected to lie in the absence of biases and heterogeneity. S.E., standard error. (PDF 53 KB) [file 259_2021_5440_MOESM1_ESM.pdf]

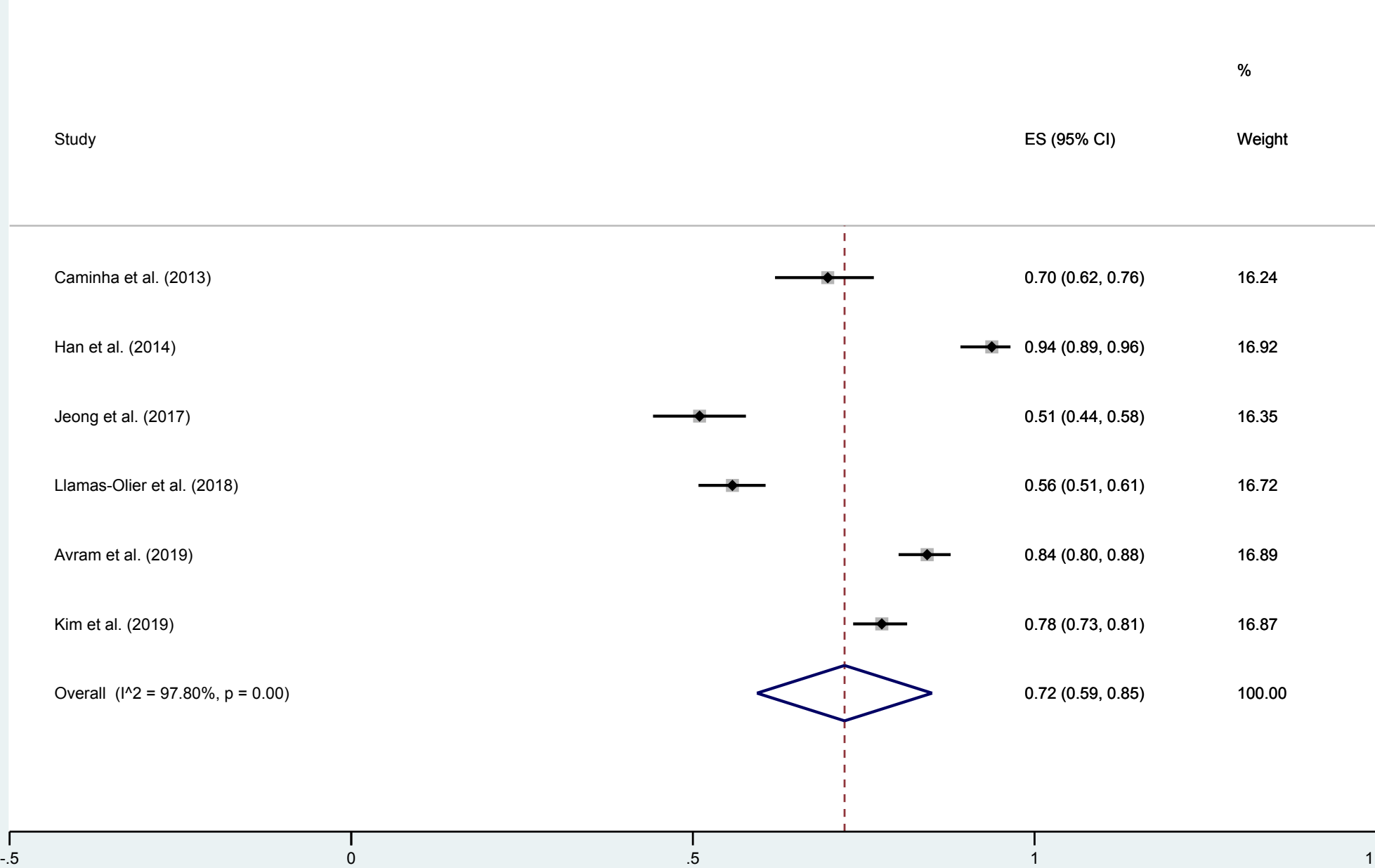

Supplement: Supplementary file 2 — Supplementary file2 Figure S2 Forest plot for the successful ablation rate after RAI therapy in the six studies considering patients treated with comparable RAI activities. Horizontal lines represent 95% confidence interval of the point estimates. The diamond represents the pooled estimate (size of the diamond = 95% confidence interval). The solid vertical line represents the reference of no increased risk and the dashed vertical line represents the overall point estimate. (PDF 55.6 KB) [file 259_2021_5440_MOESM2_ESM.pdf]

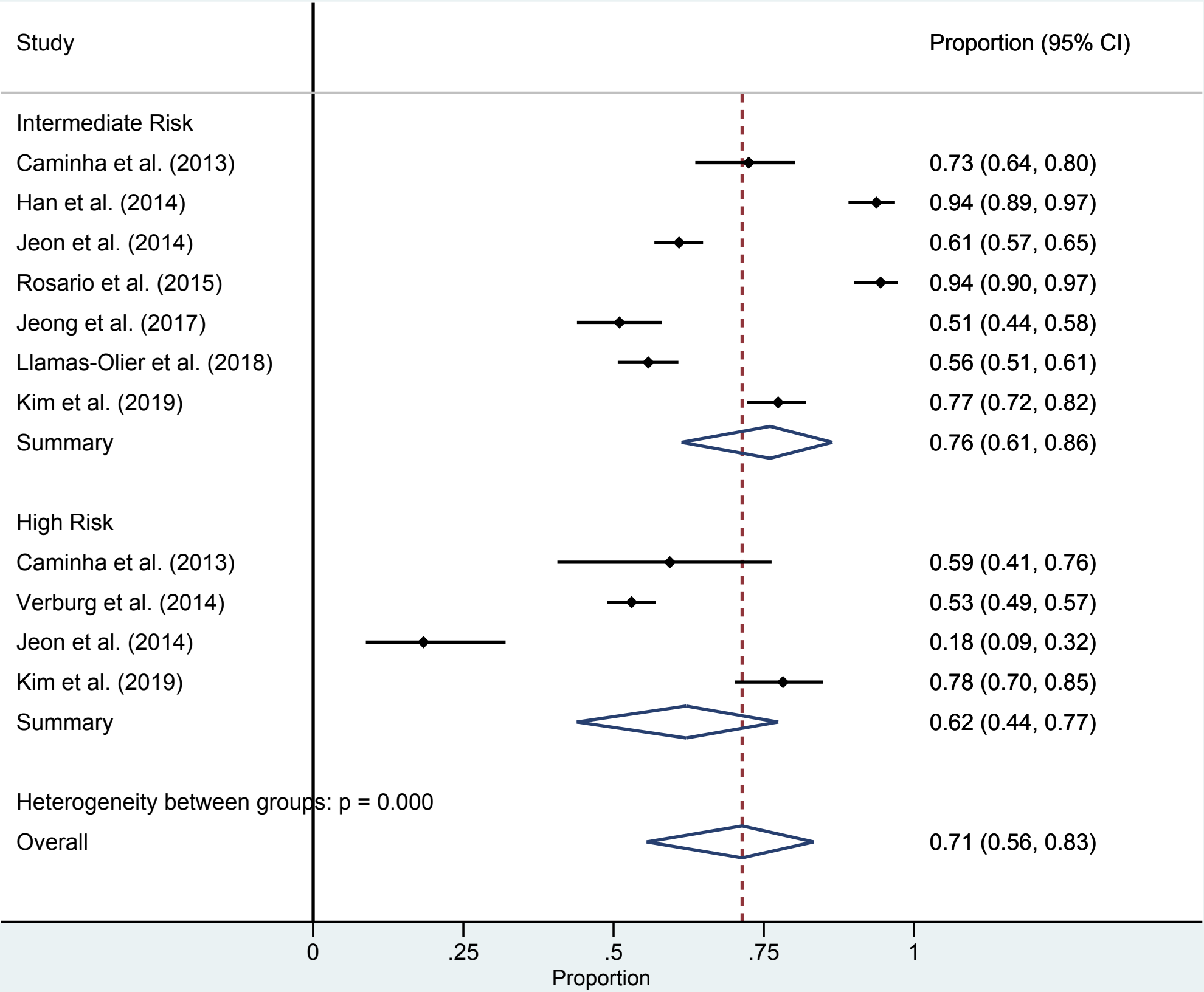

Supplement: Supplementary file 3 — Supplementary file3 Figure S3 Forest plot of relative ratios of intermediate risk patients over high-risk patients SA rates. Horizontal lines represent 95% confidence interval of the point estimates. The diamond represents the pooled estimate (size of the diamond = 95% confidence interval). The solid vertical line represents the reference of no increased risk and the dashed vertical line represents the overall point estimate. (PDF 57.7 KB) [file 259_2021_5440_MOESM3_ESM.pdf]
