# Supplementary material for: Ablation rate after radioactive iodine therapy in patients with differentiated thyroid cancer at intermediate or high risk of recurrence: a systematic review and a meta-analysis
Source: Eur J Nucl Med Mol Imaging. 2021 Jun 18;48(13):4437–44. doi: 10.1007/s00259-021-05440-x (PMC8566414; doi:10.1007/s00259-021-05440-x)
Supplement: Supplementary file 4 — Supplementary file4 (DOCX 14.6 KB) [file 259_2021_5440_MOESM4_ESM.docx]

**Leave one-out sensitivity analysis for successful ablation**

| **Study omitted** | **Successful ablation after RAI therapy** |  |
| --- | --- | --- |
| Caminha 2013 [14] | 0.71 (0.58-0.84) |  |
| Han 2014 [15] | 0.68 (0.56-0.80) |  |
| Verburg 2014 [16] | 0.73 (0.61-0.85) |  |
| Jeon 2014 [17] | 0.73 (0.60-0.85) |  |
| Rosario 2015 [18] | 0.68 (0.56-0.80) |  |
| Jeong 2015 [19] | 0.73 (0.61-0.86) |  |
| Llamas-Olier 2018 [20] | 0.73 (0.60-0.85) |  |
| Avram 2019 [21] | 0.69 (0.56-0.83) |  |
| Kim 2019 [22] | 0.70 (0.56-0.84) |  |

*RAI*, radioactive iodine
